# Discrepancies on the association between androgen deprivation therapy for prostate cancer and subsequent dementia: meta-analysis and meta-regression

**DOI:** 10.18632/oncotarget.20391

**Published:** 2017-08-22

**Authors:** Jae Heon Kim, Bora Lee, Deok Hyun Han, Kyoung Jin Chung, In Gab Jeong, Benjamin I. Chung

**Affiliations:** ^1^ Department of Urology, Stanford University Medical Center, Stanford, CA, USA; ^2^ Department of Urology, Soonchunhyang University Hospital, Soonchuhyang University Medical College, Seoul, Korea; ^3^ Department of Biostatistics, Clinical Trial Center, Soonchunhyang University Bucheon Hospital, Bucheon, Korea; ^4^ Department of Statistics, Graduate School of Chung-Ang University, Seoul, Korea

**Keywords:** androgen deprivation therapy, prostate cancer, dementia, alzheimer’s disease

## Abstract

Limited literature exists on the association between androgen deprivation therapy (ADT) for prostate cancer (PCa) and subsequent dementia and the study conclusions are in conflicts with one another. We searched several cohort databases from 1960 to 2017 for observational or prospective studies that reported on an association between ADT for PCa and subsequent dementia. A meta-analysis was performed to cumulatively determine the association between ADT and dementia including Alzheimer's disease using an incidence rate ratio (IRR), crude hazard ratio (HR), and adjusted HR. Seven studies were eligible for the meta-analysis, with the inclusion of a total of 90, 543 prostate cancer patients. The pooled overall IRR, crude HR, and adjusted HR were 1.78 [95% confidence interval (CI): 1.51–2.10)], 1.80 (95% CI: 1.05–3.10), and 1.59 (95% CI: 1.16–2.18), respectively. A meta-regression analysis showed that the crude HR was affected by both follow -up duration and lag time in the univariate model (*p* = < 0.001). However, IRR and adjusted HR were not affected by these moderators. The overall outcomes of IRR, crude HR, and adjusted HR were found to be balanced in the sensitivity analysis. A positive association was demonstrated between ADT and the subsequent incidence of dementia in this meta-analysis. Methodological difference including follow-up duration and the time lag could be related with the discrepancies.

## INTRODUCTION

Prostate cancer (PCa) is considered to be one of most common malignancies in men worldwide. Although androgen deprivation therapy (ADT) is no longer the first-line treatment for localized PCa, it is still regarded as the treatment of choice for advanced PCa due to lowering of androgen levels or stopping them to stimulate PCa cells [1, 2].

However, negative perceptions of ADT remain owing to related complications, including osteoporosis, diabetes mellitus, and cardiovascular events [3–6]. By several recent studies, early ADT is no longer actively recommended, even in advanced PCa and cases of locally invasive or metastatic cancer, as there is no notable benefit with regard to survival [7–9].

Of the reported adverse events, subsequent dementia including Alzheimer's disease (AD), is becoming an issue. The pathophysiological mechanism involved is shared in both cardiovascular disease and cognitive disorders, which has already documented in recent studies [5, 10, 11]. Recognizing the negative aspects of ADT is important because complications following ADT treatment can be irreversible. Interestingly, there is no difference in the results obtained following the administration of either continuous or intermittent ADT treatment for osteoporosis, ischemic or thrombotic events, and cognition disorders [9].

Androgen plays a pivotal role in the pathological mechanism that is explained by neuronal health and growth, conversion to estrogen, and interaction with the receptors [12–15]. It is plausible to suspect a direct or indirect causal relationship between ADT and dementia/AD in view of the fact that ADT complications extend to cognitive disorders and vascular compromise. However, conflicting conclusions have been reported in the literature on this issue [16–22].

Thus, the study objective was to establish whether or not there is an association between ADT for PCa and subsequent dementia and to highlight possible reasons for the discrepancies reported in the literature in this regard, including differences in the methodologies used and characteristics of each cohort database.

## RESULTS

### Final inclusion of subjects

**Figure 1:**
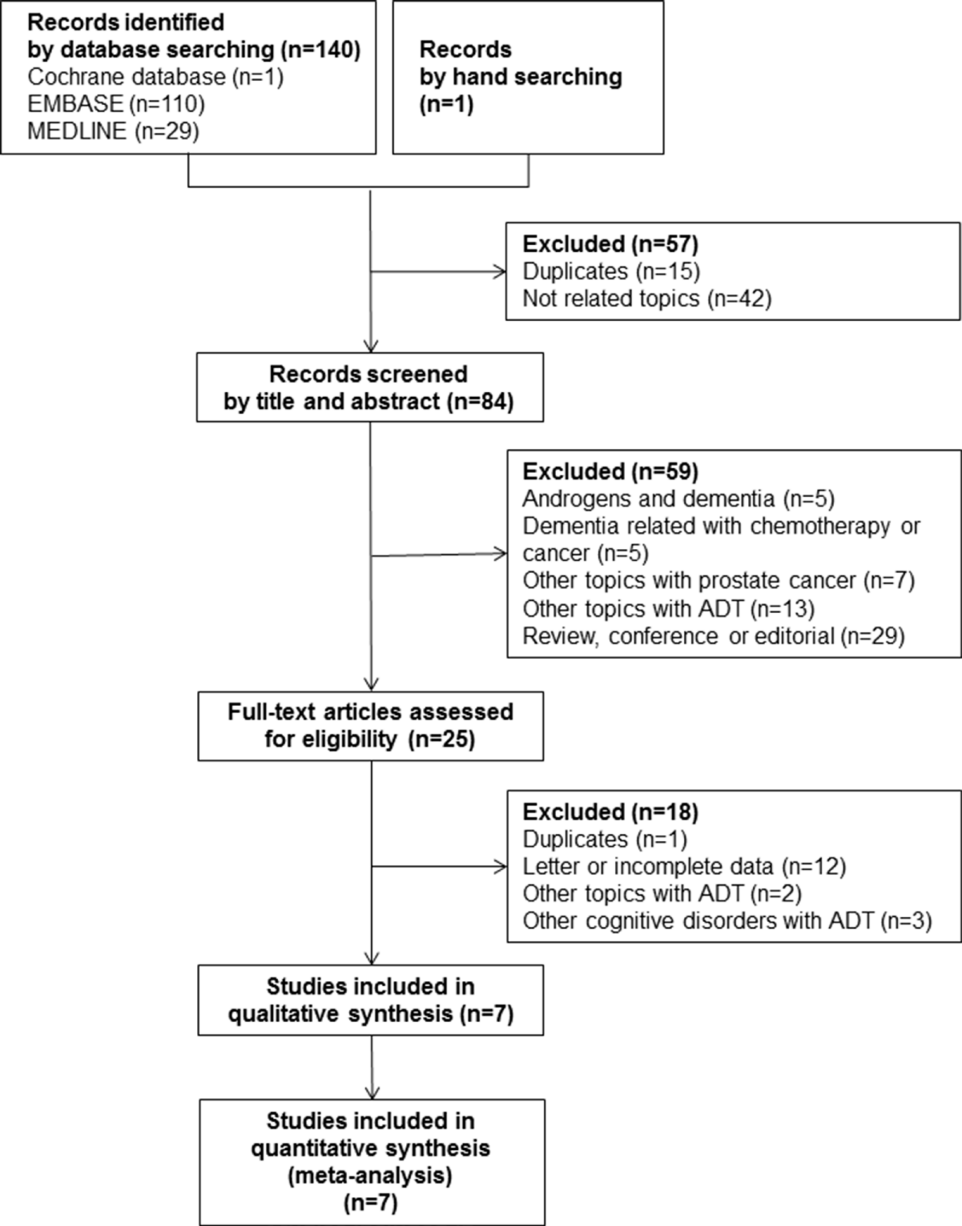
A flow diagram of the study selection process and inclusion criteria used for the meta-analysis

A total of 140 articles were identified from the electronic cohort databases in the initial search Medline (#29), Cochrane (#1), and Embase (#110). One study was added by hand searching. After eliminating 15 studies that contained overlapping data or that appeared in more than one cohort database, and after screening the titles and abstracts, 84 studies were determined to be eligible for intensive screening. Of these, 59 studies were eliminated as they were on androgens and dementia (#5), chemotherapy and dementia (#5), other prostate cancer topics (#7), other ADT topics (#13), and other study types, such as commentaries and reviews (#29). The full text of 25 studies was evaluated. Eighteen were excluded because of duplication, incomplete data (#12), and other ADT topics (#1). During data synthesis, a total of four studies were additionally excluded due to comparing intermittent ADT and continuous ADT [23], and ADT-related cognitive disorders without detailed information of HR or RR [24–26]. Finally, seven studies involving 90,543 subjects (38,307 ADT-exposed subjects and 52,236 non-exposed subjects) met the inclusion criteria. The study selection process is detailed in a flow chart (Figure 1). Details of the research duration (2016–2017) and a description of the subjects in the seven included studies [16–22] is shown in Table 1.

**Table 1:** Characteristics of studies

Author (year)	Study design	Enrollment period	Ethnicity	No. of patients			Mean Age (SD)		Definition of dementia		Study period		
				Total cohort (case/control)	Cases studied	Controls studied	Cases	Controls	Type	Code	Index date	Follow-up duration (year)	Lagging time (month)
Kao, et al. (2016)	Retrospective cohort	2001–2008	Asian	1,481/1,314	755	559	74.2 (7.9)	69.3 (10.1)	All types	290.0–290.4, 294.1, 331.0–331.2, or 331.82	The date of the first ambulatory care visit	5	-
Chung, et al. (2016)	Retrospective cohort	2001–2008	Asian	1,481/1,335	768	567	74.2 (8.0)	69.5 (10.2)	Alzheimer disease	331.0	The date of the first ambulatory care visit	5	-
Nead, et al. (2016)	Retrospective cohort	1994–2013	White or others	18,218/16,888	2397	14491	70.9 (10.8)	66.7 (10.5)	Alzheimer disease	331.0	The date of the first ambulatory care visit	2.7	6
Nead, et al. (2016)^a^	Retrospective cohort	-	White or others	18,218/6,671	1292	5379	78.9 (6.9)	77.5 (6.5)	Alzheimer disease	331.0	The date of the first ambulatory care visit	2.7	6
Nead, et al. (2017)	Retrospective cohort	1994–2013	White or others	9,455/9,272	1826	7446	69.9 (11.0)	66.2 (10.8)	All types	290.0–290.9, 331.0–331.2, or 294.1–294.21	The date of the first ambulatory care visit	3.4	6
Khosrow-Khaver, et al. (2017)	Retrospective cohort	-	White or others	52,599/30,903	15310	15393	72.8 (8.3)	68.7 (9.0)	All types	-	1 year after the date of the first prescription, or surgery date of the bilateral crhiectomy	2.3	12
Jhan, et al. (2017)	Retrospective cohort	-	Asian	28,178/24,360	15959	8401	75.48 (6.92)	74.28 (7.26)	Alzheimer disease	290.1–290.3, 294.1–294.2, and 331.0	The date of the first ambulatory care visit	4	3

a, publicated on Scientific Reports.

### Quality assessment and reporting bias

A quality assessment of the included studies was conducted using the NOS (Supplementary Table 1). A detailed description of the ADT-exposed and non-exposed cohorts was provided in all the studies. Three studies by Nead *et al*. [16–18] were not representative of the exposed cohort because national health data were not used. However, all included studies showed low risk of bias. Thus, all seven studies were evaluated for an association between the use of ADT for PCa and subsequent dementia.

### Main outcomes including incidence rate ratio, crude hazard ratio, and adjusted hazard ratio

The pooled incidence rates per 1,000 person-years for dementia/AD were 4.67 (95% CI: 3.10–7.04; I^2^ = 84.9%) in the ADT-exposed group and 2.81 (95% CI: 1.96–4.03; I^2^ = 82.3%) in the non-exposed group. The corresponding results for AD were 3.19 (95% CI: 2.13–4.79; I^2^ = 0.0%) and 1.87 (95% CI: 1.32– 2.64; I^2^ = 0.0%) (Table 2).

**Table 2:** Results of the meta-analysis

Author (year, journal)	Incidence rate per 1000 (95% CI)		IRR		Crude HR		Adjusted HR	
	In cases	In controls	Ratio (95% CI)	*p*-value	Ratio (95% CI)	*p*-value	Ratio (95% CI)	*p*-value
Dementia including Alzheimer								
Kao, et al. (2016)	2.36 (1.22–4.55)	1.86 (0.79–4.39)	1.27 (0.43–3.74)		0.90 (0.62–1.31)		1.21 (0.82–1.79)	
Chung, et al. (2016)	3.44 (2.00–5.90)	1.69 (0.69–4.14)	2.02 (0.71–5.73)		2.05 (0.64–6.52)		1.76 (0.55–5.59)	
Nead, et al. (2016)	-	-	-		-		1.66 (1.06–2.61)	
Nead, et al. (2016)^a^	2.90 (1.56–5.36)	1.90 (1.31–2.76)	1.53 (0.74–3.14)		-		2.04 (1.23–3.40)	
Nead, et al. (2017)	7.89 (5.97–10.44)	3.50 (2.84–4.31)	2.26 (1.69–3.20)		3.00 (2.33–3.87)		2.21 (1.71–2.85)	
Khosrow-Khaver, et al. (2017)	7.40 (6.55–8.35)	4.40 (3.76–5.15)	1.68 (1.38–2.05)		-		1.02 (0.87–1.19)	
Jhan, et al. (2017)	-	-	-		1.96 (1.55–2.48)		1.84 (1.32–2.57)	
Overall by random effect model	4.67 (3.10–7.04)	2.81 (1.96–4.03)	1.78 (1.51–2.10)	< 0.001	1.80 (1.05–3.10)	0.032	1.59 (1.16–2.18)	0.004
Heterogeneity, I^2^ (%)	84.9% (66.3–93.2%)	82.3% (59.2–92.3%)	0.0% (0.0–69.3%)	0.606	89.1% (74.7–95.3%)	< 0.001	82.2% (64.5–91.1%)	< 0.001
Alzheimer disease only								
Chung, et al. (2016)	3.44 (2.00–5.90)	1.69 (0.69–4.14)	2.03 (0.71–5.77)		2.05 (0.64–6.52)		1.76 (0.55–5.59)	
Nead, et al. (2016)	-	-	-		-		1.66 (1.06–2.61)	
Nead, et al. (2016)	2.90 (1.56–5.36)	1.90 (1.31–2.76)	1.53 (0.74–3.14)		-		2.04 (1.23–3.40)	
Jhan, et al. (2017)	-	-	-		1.96 (1.55–2.48)		1.84 (1.32–2.57)	
Overall by random effect model	3.19 (2.13–4.79)	1.87 (1.32–2.64)	1.67 (0.92–3.03)	0.09	1.96 (1.56–2.47)	< 0.001	1.83 (1.45–2.30)	< 0.001
Heterogeneity, I^2^ (%)	0.0% (NA)	0.0% (NA)	0.0% (NA)	0.656	0.0% (NA)	0.941	0.0% (0.0–0.0%)	0.949

NA, not available; IRR, incidence rate ratio; HR, hazard ratio; CI, confidence interval.

a, publicated on Scientific Reports.

**Figure 2:**
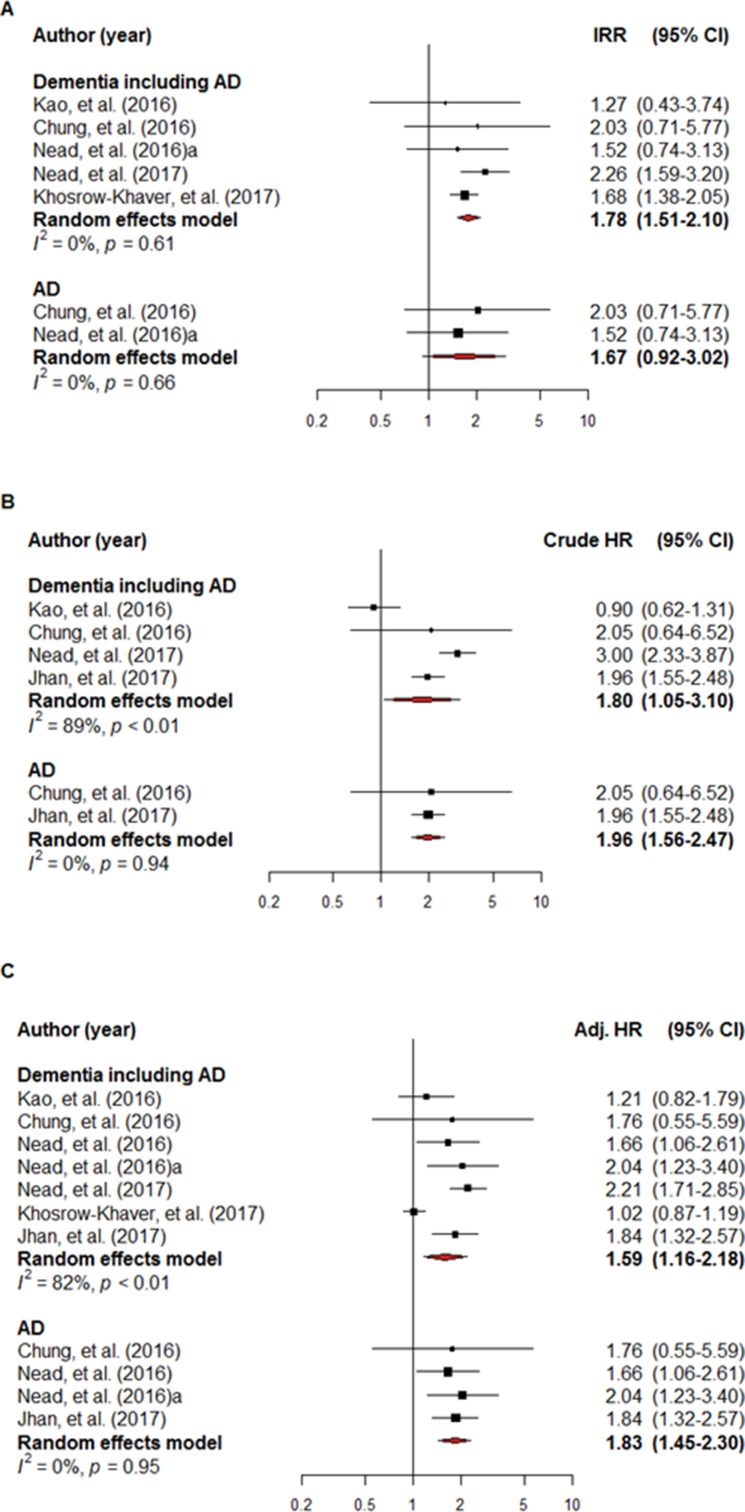
A forest plot of the overall incidence rate ratio (**A**), crude hazard ratio (**B**) and adjusted hazard ratio (**C**). The black square signifies the weighted mean of each estimate. All data pertain to continuous outcomes.

The IRR of the individual studies ranged from 1.53–2.26 and a statistically significant result (i.e., if the 95% CI crossed 1) was not reported in three of these five studies (60.0%) when a comparison was made between the ADT-exposed and non-exposed groups. The pooled IRR data indicated that ADT exposure was a high risk factor for dementia/AD (IRR = 1.78, 95% CI: 1.51–2.10; I^2^ = 0.0%). ADT exposure was also shown to be a high risk factor for AD (IRR = 1.67, 95% CI: 0.92–3.03; I^2^ = 0.0%), although this finding was not statistically significant (Figure 2A).

The crude HR for dementia/AD was reported in four studies. A non-statistically significant HR (i.e., if the 95% CI crossed 1) and overall crude HR for dementia/AD was 1.80 (95% CI: 1.05–3.10; I^2^ = 89.1%) in two of the eligible four studies (50.0%). The overall crude HR for AD in the two studies that met the inclusion criteria was 1.96 (95% CI: 1.56–2.47; I^2^ = 0.0%) (Figure 2B).

Adjusted hazard ratios (HRs) for dementia/AD were reported in all seven studies and a non-statistically significant HR was reported in three of them (42.9%). Overall, ADT exposure was associated with an adjusted HR of 1.59 (95% CI: 1.16–2.18; I^2^ = 82.2%) for dementia/AD. Overall, the adjusted HR for AD in four of the studies that met the inclusion criteria was 1.83 (95% CI: 1.45–2.30; I^2^ = 0.0%) (Figure 2C).

**Figure 3:**
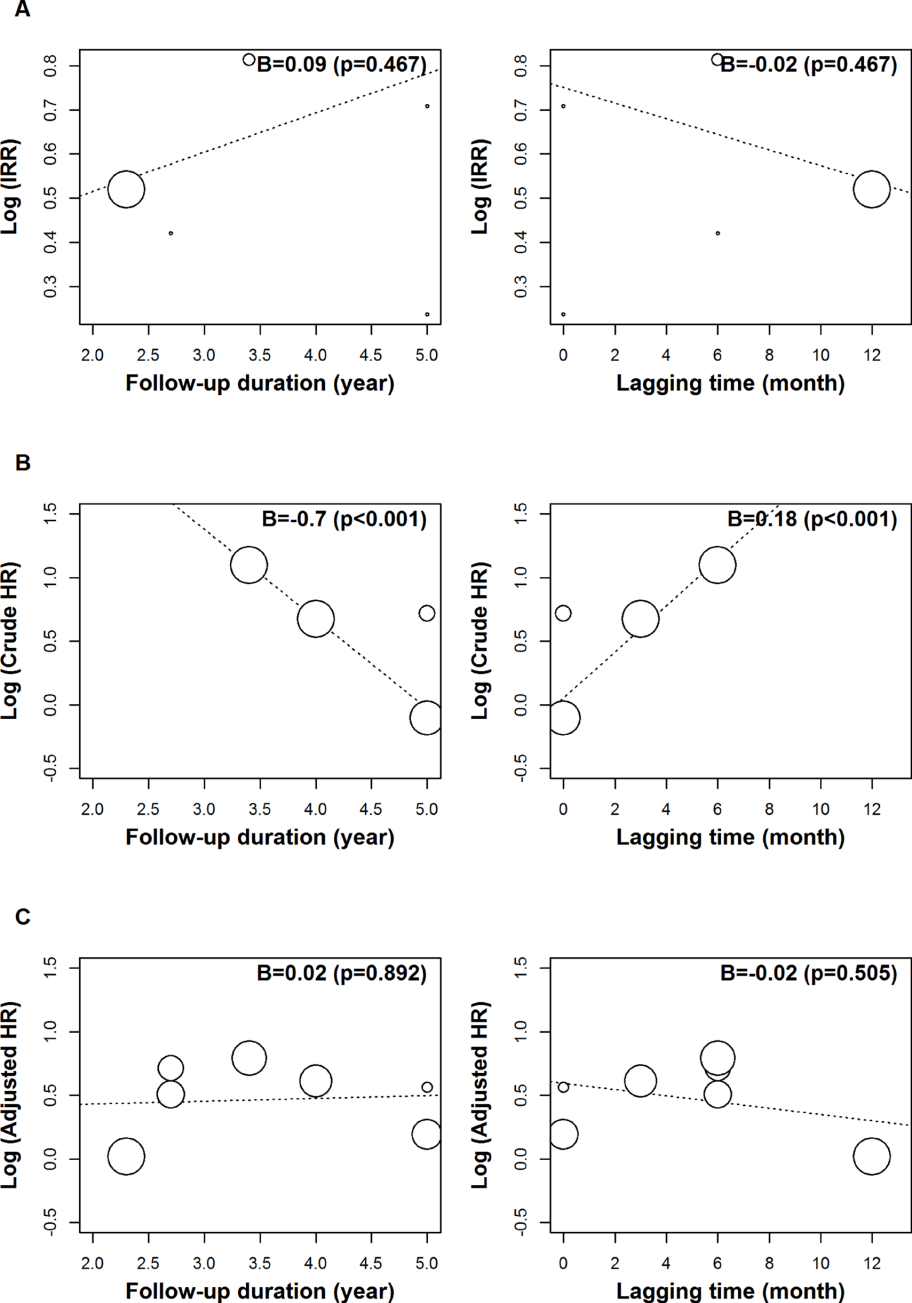
A sensitivity analysis of the incidence rate ratio, crude hazard ratio, and adjusted hazard ratio

**Figure 4:**
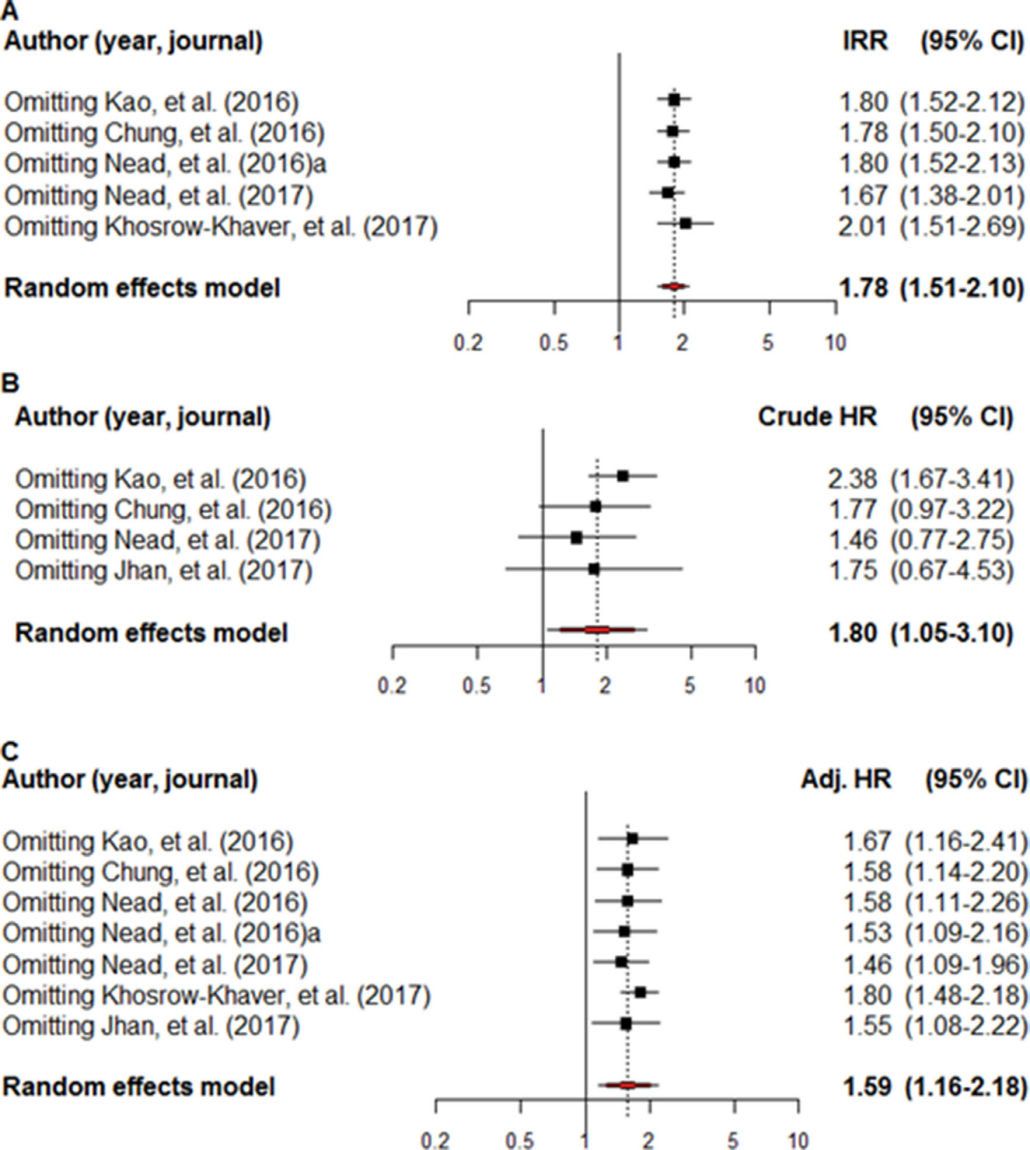
A meta-regression analysis of the incidence rate ratio (**A**), crude hazard ratio (**B**) and adjusted hazard ratio (**C**), using moderators of follow-up duration and lag time. The black square signifies the weighted mean of each estimate. All data pertain to continuous outcomes.

The robust effects of the IRR, crude HR, and adjusted HR were demonstrated on sensitivity analysis (Figure 3). The respective lowest and highest reported IRRs were 1.67 and 2.01, with corresponding figures of 1.47 and 2.38 for crude HR, and 1.46 and 1.80 for adjusted HR. Follow-up duration and lagging time were shown to be significant moderators of crude HR on meta-regression and univariate analysis (*p* = < 0.001) (Table 3). However, these moderators were not significantly related to the IRR and adjusted HR (Figure 4). A different outcome as a result of ethnicity was indicated, based on the overall IRR, following subgroup analysis. The overall IRR was 1.62 (95% CI: 0.76–3.43) in Asians and 1.81 (95% CI: 1.49–2.19) in the non-Asians (Supplementary Figure 1). Overall, a different outcome, as a result of ethnicity, was demonstrated using the adjusted HR. The overall adjusted HR was 1.54 (95% CI:1.14–2.09) in Asians and 1.63 (95% CI: 1.02–2.62) in non-Asians.

**Table 3:** Meta-regression analysis

Variable	For Log (IRR)		For Log (Crude HR)		For Log (Adjusted HR)	
	B (95% CI)	*p*-value	B (95% CI)	*p*-value	B (95% CI)	*p*-value
F/U duration (year)	0.09 (−0.15, 0.33)	0.467	−0.70 (−0.98, −0.43)	< 0.001	0.02 (−0.30, 0.35)	0.892
Lagging time (month)	−0.02 (−0.07, 0..03)	0.467	0.18 (0.10, 0.26)	< 0.001	−0.02 (−0.10, 0.05)	0.505

IRR, incidence rate ratio; HR, hazard ratio; F/U, follow up; B: the regression coefficient of linear regression analysis.

## DISCUSSION

As currently only limited literature with conflicting results exists on the association between androgen deprivation therapy (ADT) for prostate cancer (PCa) and subsequent dementia, the primary objective of this study was to fully investigate this association to understand possible reasons for the discrepancy, including methodologic differences and cohort characteristics. To address the divergence of opinion, our meta-analysis found a positive association between ADT and subsequent dementia, based on the IRR, crude HR, and adjusted HR. To overcome any heterogeneity issues, sensitivity and subgroup analyses were also performed and were shown to support the balanced and sound results obtained by the meta-analysis. Although a conclusive outcome was established regarding an association between ADT for PCa and subsequent dementia following our meta-analysis, the reasons for these discrepancies in the literature should be elucidated.

In the literature, a direct causal relationship between ADT and the risk of dementia could not be established due to the nature of observational studies. Hence, the results of observational studies should be interpreted cautiously. Only three clinical studies have investigated this issue [16, 19, 20] and they were all observational studies. Discrepancy in the incidence of AD (and associated HRs) has been reported in the literature regarding subsequent dementia and AD following ADT for PCa [17, 18, 21, 22].

There were numerous methodological differences in the methodology used including definitions for the cohort, ADT exposure, and event occurrence that could, in turn, account for differences in conclusions. These differences could relate directly to selection and misclassification bias, thus explaining the inconsistent results among the included studies. Khosrow-Khavar et al. [19] investigated a possible link between ADT and the risk of dementia in their large population-based cohort study and concluded that there was no such association. Interestingly, Nead et al. [16] investigated the same issue and reported the exact opposite.

We found that lag time was a significant moderator of crude HR in our study, which implies that the lag period selected could affect the HR. Khosrow-Khavar and others [19–21] set specific lag times. In the case of Khosrow-Khavar et al. [19], a fixed lag period of a year was set and the implementation of time-dependent exposure. By using this method [19–21], misclassification and immortal bias may have been avoided. However, further bias could result from the adoption of a fixed lag period based on biological knowledge or expert opinion, which could lead to a null hypothesis. If the estimation of a lag period is not calculated directly, it could also result in a null hypothesis, owing to bias [27, 28]. Some, like Nead et al. [16, 19] did not clearly define a lag period, but did apply a six-month latency period for the development of dementia.

Follow-up duration was also shown to be a significant moderator of crude HR. Nead et al. [16] stated it to be 7.9% in the ADT-exposed group and 3.5% in the non-exposed group. In our study, the incidence rate was 7.4% and 4.4%. If a longer latency period causes a decrease in the crude incidence rate, the true incidence rate or crude HR, without the adoption of a lag period, could be much higher than the currently reported incidence rate.

Heterogeneity both in defining the ADT exposed group and in the ADT treatment administered, could affect outcomes. These could be due to the practice styles and characteristics of the healthcare providers which could influence the pattern of ADT treatment. This might be an important reason why there was an increased number of patients who were exposed to ADT in the study of Khosrow-Khavar et al. Although at-risk patients and the time interval used are more important than the number of patients, crude HR could be decreased by reducing the cumulative HR in the ADT-exposed group (numerator) when a relatively short follow-up period is used. If this is true, a robust ADT treatment approach is required in order to obtain an enhanced understanding of these observational studies.

Although we, drew a scientific conclusion based on the results of a meta-analysis, meta-regression, and sensitivity analysis, there are several limitations exist. First of all, included studies are observational studies in nature, hence, limitations exist in drawing a causal relationship between ADT and subsequent dementia. Although our study demonstrated a significant association between ADT and subsequent dementia, these results do not support the decreased use of ADT in advanced PCa. However, our study underscores the potential complications including dementia during ADT for advanced PCa. Another limitation is that there is no clear discrimination of continuous ADT from intermittent ADT in each included study. Several recent studies show that continuous ADT has limited benefits compared to intermittent ADT, including a marginally superior survival rate of approximately 5% in selected PCa patients [7, 9]. However, recently, it has also been demonstrated that there are no differences in the risk of subsequent complications developing with the use of either continuous or intermittent ADT [7, 9]. It has also been shown that impaired cognitive function could not be restored by testosterone therapy [29], implying that any adverse effects relating to cognitive function are irreversible. Future studies are warranted on the prevention of subsequent dementia following ADT treatment.

A scientific definition of an ADT cohort, the indications for ADT treatment, and the incidence of dementia (using an inner validation tool) should be established in future studies. The follow-up duration should be increased and the strategy used to determine the lag or latency period must involve a direct scientific calculation. Future studies should also include information on detailed cancer staging and should categorize ADT types. This is because ADT has varied complex effects on the hypothalamic-pituitary-gonadal axis. Using this information, a valid HR for ADT according to cancer stage could be determined and compared with the survival benefit conferred by ADT.

In summary, ADT remains the mainstay treatment for advanced PCa; however, it is also important to recognize its potential side-effects, one of which is dementia. Dementia/AD is an important disease that impacts considerably on public health, especially as its incidence increases with advancing age. However, the current evidence in observational studies about an association between ADT and subsequent dementia is limited. Considering the difficulty in performing randomized controlled trials on this issue, Our goal was to obtain conclusive, informative evidence by a meta-analysis. We established a positive association between ADT and subsequent dementia, according to our meta-analysis, the heterogeneity of the results between the studies could be due to differences in the methodologies used, study parameters, characteristics of cohort database, and aging phenomenon.

## MATERIALS AND METHODS

A systematic review and meta-analysis without language restrictions was carried out in accordance with the Meta-analysis Of Observational Studies in Epidemiology (MOOSE) guidelines [30].

### Searching strategies

A review was conducted of observational or prospective clinical studies reporting on ADT for PCa/dementia. Studies were included if they met the criteria of the inclusion of male patients with PCa who had or had not been exposed to ADT. Main outcomes included hazard ratio, relative risk, and incidence rate for any type of subsequent dementia.

A cross-search of the relevant literature until January 2017 was performed using Medline. An optimally sensitive Cochrane Collaboration search strategy was applied using various medical subject headings, including “prostatic neoplasms”, “Alzheimer's disease”, “dementia”, “androgen antagonists”, “androgen receptor antagonists”, “leuprolide”, “gonadotropin-releasing hormone”, and “triptorelin pamoate”. Key search terms with natural language headings were “PCa”, “prostate tumor”, “prostate neoplasm”, “prostate adenocarcinoma”, “Alzheimer's disease”, “dementia”, “androgen antagonist”, “antiandrogen”, “androgen receptor antagonist”, “androgen deprivation therapy”, “leuproelin”, “gonadotropin-releasing hormone”, “gonadorelin”, “triptorelin”, and “degarelix”. The search extended to titles and abstracts, Embase (from January 1980 to December 2016), and the Cochrane Library. There were no language restrictions. Data were recorded on the first author, publication year, ethnicity, sample size, age range of the participants, definition of the cohort, definition of ADT exposure, definition of event occurrence, investigated incidence rates, and crude or adjusted HRs. Study inclusion criteria was including hazard ratio or relative risk.

### Data collection and analysis

Initial screening of the electronic cohort databases for studies was based on the information provided in the titles and abstracts. The screening was performed by two researchers and complete study reports were reviewed. The full text of the articles was reviewed for further information and clarification in cases of insufficient data. Final selection was determined by discussion. References and data for each included study were carefully cross-checked for overlapping of data and to ensure the integrity of the investigation.

### Primary outcomes

Risk measurements for dementia/AD, according to exposure to ADT, were the primary study outcomes and included incidence rate ratio (IRR), crude hazard ratio (HR), and adjusted HR. To calculate the IRRs, the incidence rate of dementia/AD in each study was computed by dividing the number of incidents of dementia/AD by the total number of person-years of observation. If the total number of person-years of follow-up was not stated, it was calculated by multiplying the number of patients by the mean duration of follow-up. The corresponding information for AD was similarly calculated.

### Assessment of the risk of bias

The methodological quality of the studies was determined and the data extraction was carried out independently by two reviewers. Discrepancies were resolved by consensus. Risk of bias was assessed using the Newcastle-Ottawa Scale (NOS) for assessing the quality of nonrandomized studies in met-analysis [31]. Bias risk was determined to be low, moderate or high by calculating the sum of the scores.

### Statistical analysis

The incidence rates of dementia/AD according to ADT exposure were pooled according to the DerSimonian and Laird method of estimating τ^2^ using inverse variance weights after log-transforming the study-specific incidence rate per 1000 person-years. In a comparison of the ADT exposed and non-exposed groups, the risk measurements (including IRRs and HRs analyzed on the log scale) were weighted by the inverse of their corresponding variance to obtain the pooled estimates using 95% confidence intervals (CIs). Since clinical heterogeneity was expected with regard to the study location, cohort database cohort, and methodology used, a random effects model was applied using the method of DerSimonian and Laird.

Meta-regression analysis was conducted for IRR, crude HR, and adjusted HR to determine any potential moderators. Variability in the effect sizes was analyzed owing to differences between the moderators (e.g., follow-up duration and lagging time). A restricted maximum likelihood estimator was used to evaluate the true effects of the between-study variance. Subgroup analysis was performed to evaluate the effects of ethnicity.

Heterogeneity across the studies was appraised using *p*-values, and Q and I^2^ statistics. The I^2^ results were categorized as follows: *Not important:* < 30%; *Moderate:* 30–50%; *Substantial:* 50–75%; *Considerable:* > 75%.

To assess the effect of individual studies on the pooled estimates, sensitivity analysis was conducted by re-estimating the results by omitting one study at a time. Publication bias was not assessed owing to the small number of studies included. The analysis was performed using R^®^ (version 3.3.2) (The R Foundation for Statistical Computing, Vienna, Austria).

## SUPPLEMENTARY FIGURE AND TABLE


