# Supplementary material for: Discrepancies on the association between androgen deprivation therapy for prostate cancer and subsequent dementia: meta-analysis and meta-regression
Source: Oncotarget. 2017 Aug 22;8(42):73087–97. doi: 10.18632/oncotarget.20391 (PMC5641194; doi:10.18632/oncotarget.20391)
Supplement: Supplementary file 1 [file oncotarget-08-73087-s001.pdf]

Discrepancies on the association between androgen deprivation therapy for prostate cancer and subsequent dementia: meta-analysis and meta-regression

SUPPLEMENTARY MATERIALS

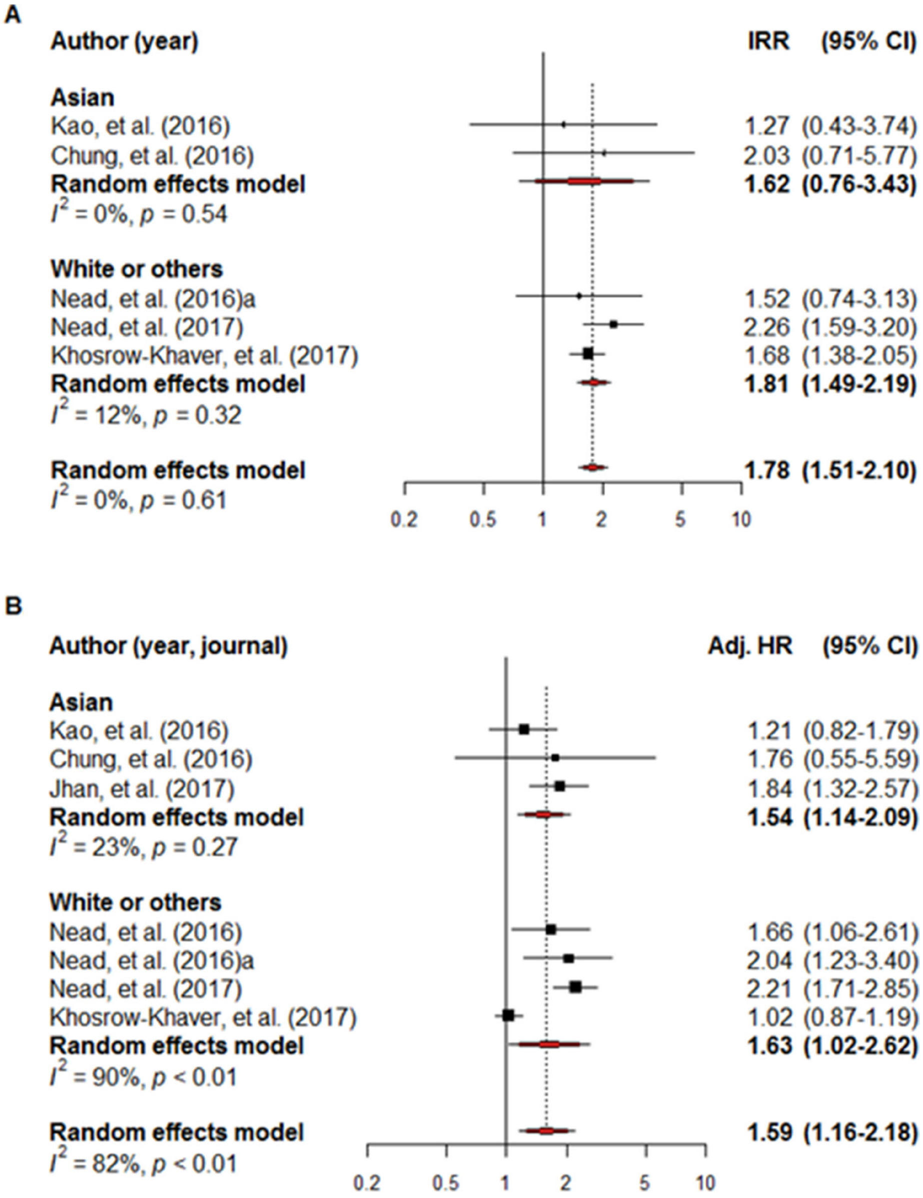

Supplementary Figure 1: A subgroup analysis of the incidence rate ratio and adjusted hazard ratio according to ethnicity.

Supplementary Table 1: Quality assessment of included studies

Author (year, journal)	Selection				Comparability	Outcome			Risk of bias
	Representativeness of the exposed cohort	Selection of the non-exposed cohort	Ascertainment of exposure	Demonstration that outcome of interest was not present at start of study	Comparability of cohorts on the basis of the design or analysis	Assessment of outcome	Was followup long enough for outcomes to occur?	Adequacy of follow up of cohorts	No. of stars (high/moderate/low)
Kao, et al. (2016)	*	*	*	*	*	*	*	*	8 (low)
Chung, et al. (2016)	*	*	*	*	*	*	*	*	8 (low)
Nead, et al. (2016)		*	*	*	**	*	*	*	8 (low)
Nead, et al. (2016)[a]		*	*	*	**	*	*	*	8 (low)
Nead, et al. (2017)		*	*	*	**	*	*	*	8 (low)
Khosrow-Khaver, et al. (2017)	*	*	*	*	**	*	*	*	9 (low)
Jhan, et al. (2017)	*	*	*	*	**	*	*	*	9 (low)

Each asterisk represents one star in the Newcastle-Ottawa Scale system. The maximum number of stars is 2 for comparability and 1 for each of the other categories, for a total of up to 9 stars.
a, publicated on Scientific Reports.
